# Targeted Acid-Sensing Ion Channel Therapies for Migraine

**DOI:** 10.1007/s13311-018-0619-2

**Published:** 2018-03-16

**Authors:** Nazia Karsan, Eric B. Gonzales, Gregory Dussor

**Affiliations:** 1 Headache Group, Department of Basic and Clinical Neuroscience, Institute of Psychiatry, Psychology and Neuroscience, Kings College London, Denmark Hill, London, SE5 9PJ UK; 2 TCU and UNTHSC School of Medicine (applicant for LCME accreditation), Department of Medical Education, 3500 Camp Bowie Blvd., Fort Worth, TX 76107 USA; 3 School of Behavioral and Brain Sciences, The University of Texas at Dallas, 800 West Campbell Road, BSB-14, Richardson, TX 75080 USA

**Keywords:** Migraine, ASICs, Headache, Cortical spreading depression, Therapeutics, Ion channels

## Abstract

Acid-sensing ion channels (ASICs) are a family of ion channels, consisting of four members; ASIC1 to 4. These channels are sensitive to changes in pH and are expressed throughout the central and peripheral nervous systems—including brain, spinal cord, and sensory ganglia. They have been implicated in a number of neurological conditions such as stroke and cerebral ischemia, traumatic brain injury, and epilepsy, and more recently in migraine. Their expression within areas of interest in the brain in migraine, such as the hypothalamus and PAG, their demonstrated involvement in preclinical models of meningeal afferent signaling, and their role in cortical spreading depression (the electrophysiological correlate of migraine aura), has enhanced research interest into these channels as potential therapeutic targets in migraine. Migraine is a disorder with a paucity of both acute and preventive therapies available, in which at best 50% of patients respond to available medications, and these medications often have intolerable side effects. There is therefore a great need for therapeutic development for this disabling condition. This review will summarize the understanding of the structure and CNS expression of ASICs, the mechanisms for their potential role in nociception, recent work in migraine, and areas for future research and drug development.

**Electronic supplementary material:**

The online version of this article (10.1007/s13311-018-0619-2) contains supplementary material, which is available to authorized users.

## Introduction to Migraine

Migraine is a common and disabling headache condition affecting millions of people worldwide [1]. The World Health Organization (WHO) classifies migraine as the sixth most disabling disorder globally [1], with a total prevalence of 15 to 18%, regardless of which country is surveyed [2]. Typically, migraine tends to affect people during their peak working years so there is significant disability and subsequent socioeconomic impact associated with the condition [3].

Migraine is classified as a disabling disorder of moderate to severe throbbing head pain, with each attack lasting 4 to 72 h, associated with nausea and/or vomiting and other sensory sensitivities [4]. Despite migraine being well recognized as a disorder of head pain among the general public, there are a wide range of nonheadache symptoms which can be associated with migraine; thus, it has become increasingly accepted as a central disorder of sensory processing; this creates a condition in which usual sensory stimuli such as light, sound, touch, and smell are perceived as more intense or bothersome, or that there is a problem with the modulation of such stimuli by the migraine brain [5]. Additionally, there can be difficulties processing such stimuli, in which the brain is unable to block “noise” of nonuseful stimuli such as peripheral conversation, a television that has been left on, or bright lighting, and migraineurs can find managing excessive amounts of such stimulation particularly difficult, for example, in loud concerts or crowds, or in flashing lighting such as at a party. Such difficulties with sensory stimulation, in some cases, are also present interictally in the absence of head pain [6], or indeed in the premonitory or postdrome phases of the attack, in which these symptoms are present without migrainous headache [7, 8]. Migraine may therefore be viewed as a primary brain disorder, in which the brain is abnormally sensitive to sensory stimulation, be that via touch, movement, light, sound, or smell, as well as subcortical and cortical brain dysfunction, so that activation of the process leads to a cascade of further neuronal and vascular dysfunction within the brain, mediating pain as well as other associated symptoms.

Migraine is also a disorder of trigeminovascular activation; that is, it includes activation of a pathway between sensory afferents located in the meninges and vessel walls in the cranium (the only pain sensate structures within the cranium) and neck which transfer nociceptive information through the trigeminal ganglion, cervical ganglion, and greater occipital nerve into the brain, where this information converges in the trigeminocervical complex (TCC) within the pons. From here, nociceptive signaling and modulation occur through various subcortical structures, including the rostroventral medulla (RVM), the locus coeruleus (LC), periaqueductal gray (PAG), hypothalamus, and thalamus, and eventually to the cortex for the sensory processing of pain. There is also a reflex connection from the TCC through the sphenopalatine ganglion (SPG) to the cranial vasculature, and it is thought that this pathway is likely to mediate some of the cranial autonomic symptoms that can accompany a migraine attack [9]. Despite historical views about migraine as a vascular headache and this hypothesis explaining the clinical response to triptans and dihydroergotamine as the most effective migraine abortives (mediating their action via vasoconstriction [10]), it has become increasingly clear that although the blood vessels do play a part in the disorder, migraine is much more a neural rather than vascular brain disorder, with several cortical and subcortical brain areas and ascending and descending pathways involved.

Despite decades of research into migraine, there remains a paucity of effective acute or preventive treatments available to treat the disorder. Most of the preventive treatments available were initially developed for the treatment of other conditions such as epilepsy, depression, or hypertension, and their helpful effect in migraine was noticed by chance. This means that the treatments are nonspecific and untargeted to migraine mechanisms, complicating these drugs by adverse effect profiles and tolerability issues as well as suboptimal efficacy. With regards to acute treatments, there have been no real breakthroughs in targeted abortive development since the triptans in the early 1990s [11–13], which, despite excellent efficacy in a proportion of patients, do not work for everyone [14], can be complicated by headache recurrence [15], and can cause vascular effects which are often unfavorable and contraindicate their use in anyone with preexisting cardiovascular, cerebrovascular, or peripheral vascular disease, and in anyone over the age of 65 [16]. The drugs also carry a limited license in children, and only intranasal sumatriptan is currently available for use in this group [17]. Medication overuse is another noted complication of frequent triptan use and can lead to further difficulties treating the migraine [18]. Other acute treatments used come from other pain conditions, and largely consist of nonsteroidal anti-inflammatory drugs (NSAIDs) and combination drugs including those containing opioids, which pose their own problems.

There is therefore a therapeutic gap in migraine, and an ever-increasing need to develop targeted therapeutics for the condition, to limit its impact and burden without adverse effects. This can only be achieved through furthering understanding of the neurochemical systems and neurobiology of the disorder, to identify novel therapeutic targets within the migraine system, to ideally abort and/or prevent pain onset at all, and to treat associated migraine symptomatology.

## Introduction to Acid-Sensing Ion Channels

Acid-sensing ion channels are a family of ion channels which were first cloned in 1997 [19]. There are four members, ASIC1 to 4 (with several splice variants), three of which (all except ASIC4) are sensitive to changes in pH [20] (see Table 1). The channels are permeable to cations such as sodium, potassium, and in some cases calcium, and they are activated by low pH, both intracellular and extracellular [21, 52, 53]. Additionally over recent years, there has been the suggestion that the channels can also be modulated by endogenous and external modulators [20]. The channels can detect and open at ranges of pH between 4.0 and 8.0 [52, 54] (ASIC3 in humans is sensitive to alkaline pH [52]). They are expressed throughout the human nervous system, both in the brain and spinal cord [55, 56]. The most broadly expressed channel within the central nervous system is ASIC1 [55], particularly ASIC1a. ASIC1a is widely expressed within the mouse brain, with particular expression within the amygdala, hippocampus, PAG, caudate, putamen, olfactory bulb, cerebral cortex layer, and cerebellum [56, 57], as well as in the spinal dorsal horn neurons in rats [22, 58]. Most ASIC subtypes are expressed on primary sensory neurons and are likely to be important for pain and mechanosensation from the skin [23]. ASIC1 and ASIC3 are highly expressed in dorsal root ganglia and in peripheral sensory neurons which supply visceral organs such as the heart and bowel as well as in muscles and joints [59–61]. See Boscardin et al. [62] for a recent review.

**Table 1:** ASIC channels and subunits, and their localization and potential functions and contributions to human disease

ASIC channel type	pH sensitivity (pH_50_) [21]	Predominant localization	Likely cellular localization	Potential function	References
ASIC1A	5.8–6.8	Central and peripheral nervous systems	Axons, cell bodies, dendrites	Cell death following injury, epilepsy, pain signaling, neuroinflammation, neurodegeneration, fear behavior	[21–35]
ASIC1B	6.1–6.2	Peripheral nervous system	Axons, cell bodies		[21, 36]
ASIC2A	4.5–4.9	Central and peripheral nervous systems	Axons, cell bodies, dendrites	Modulate ASIC1, ASIC2b, and ASIC3 function, autonomic circulatory control	[21, 37–40]
ASIC2B	Associates with other channels to have pH sensitivity	Central and peripheral nervous systems	Axons, axons terminals, cell bodies	Modulate ASIC1 function, autonomic circulatory control, acidosis-induced neuronal death	[37, 38, 41]
ASIC3	6.4–6.6	Peripheral and central nervous systems	Axons, axons terminals, cell bodies	Pain signaling, fear conditioning with exogenous expression, anxiety and aggression	[21, 42–50]
ASIC4	Not pH sensitive	Central nervous system		Fear and anxiety through ASIC1a antagonism	[51]

Maintenance of a physiological acid–base balance of cellular environments is important for normal cellular function [20]. In certain disorders, tissue acidosis can follow a range of pathological processes such as tissue ischemia and inflammation [63]. It is thought that ASICs are involved in responding to this fall in extracellular pH in such conditions, and thereby contribute to pathological mechanisms and cell death in response to acidosis [24, 64]. It is postulated that on peripheral neurons, the channels are involved in sensory function such as mechanosensation and pain sensation [65, 66], and centrally they are likely to be involved in synaptic plasticity (with channel activation through increased proton levels caused by frequent synaptic activity) [25, 56, 67, 68]. Their wide expression within the central nervous system and their role in mediating cell response to acidosis have contributed to their suggested role in human diseases, including epilepsy [26], stroke, ischemic injury [69, 70], and traumatic brain injury [71], as well as several nociceptive states [72, 73]. Through this understanding, increased research around their expression, roles, and pharmacology has subsequently led their emergence as a potential therapeutic target for such neurological disorders. Additionally, it is increasingly being recognized that they may contribute to migraine given their role in several neuronal processes linked to the disorder within the central and peripheral nervous systems.

## Molecular Structure of ASICs

**Fig. 1:**
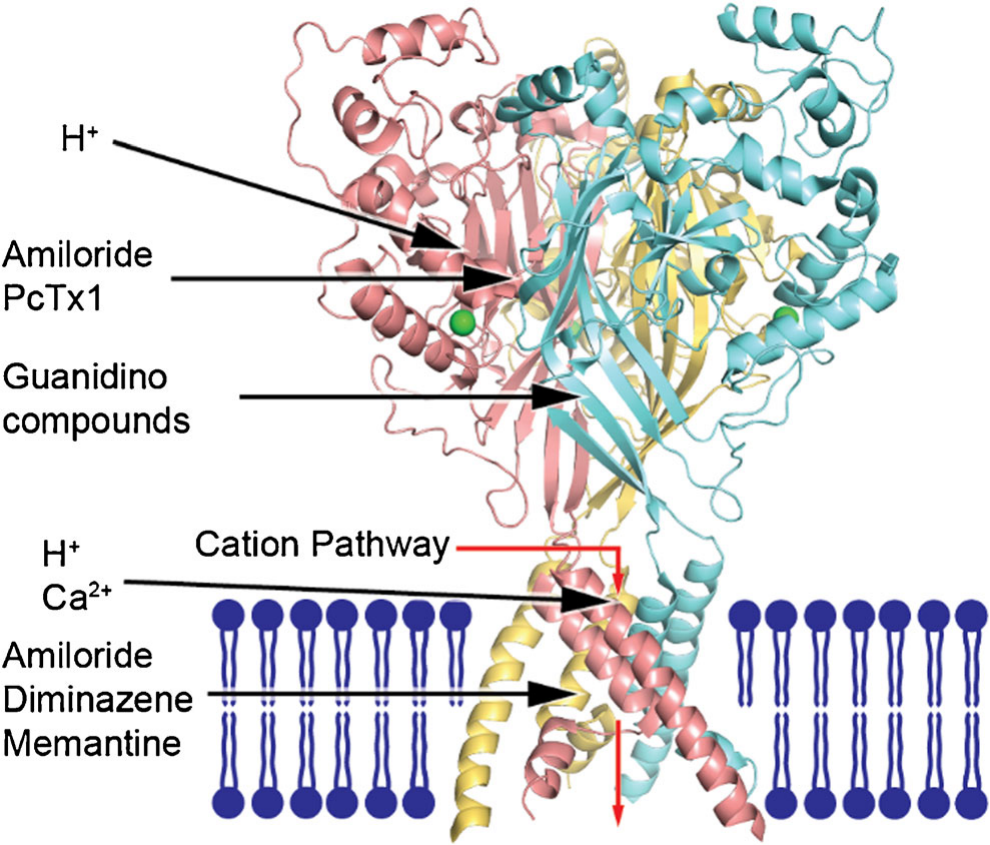
Structure of an acid-sensing ion channel (in particular chicken ASIC1)

ASICs are either Na^+^- or Ca^2+^-permeable ion channels, are formed of three subunits, and are a part of the wider family of ENaC/degenerin epithelial channels, which are involved in Na^+^ homeostasis [74] (see Fig. 1).

Although there are four main types of channel, ASIC1 to 4, subunits including ASIC1a, ASIC1b, ASIC2a, and ASIC2b also exist as splice variants, and these can form heteromultimeric channels with different functions (see Table 1). The channels are composed of three subunits that form a ligand-gated protein structure [75]. The ASIC subunits among different species are highly conserved, and human ASIC2 sequences are 99% identical to rat ASIC2. This suggests that the protein plays an important role in neuronal function across different species.

The channel subunits are composed of 500 to 560 amino acids each, and two transmembrane domains (which line the pore of the channel) [76] with a relatively short cytoplasmic section, with 35 to 90 amino acids, with a longer extracellular domain, consisting of over 300 amino acids [77]. The proton ligand binds to the extracellular domain, which is organized in a three-dimensional structure [76]. The extracellular domain is composed of 12 beta sheets and 7 alpha helices, made of several pairs of amino acids [76]. The two long alpha helices form the transmembrane domains lining the ion pore [76]. The alpha-helix of the second transmembrane domain loosens near the selectivity filter. After the selectivity filter, the remainder of the second transmembrane domain alpha-helix fills in the lower portion of the second transmembrane domain of a neighboring subunit (see Fig. 1). The extracellular domain contains an acidic section, replete with acidic amino acids, which is involved in response to extracellular acid and response to other extracellular modulators [78] (Fig. 1).

The knowledge of this structure comes from characterization of chicken ASIC1 (PDB code 4NYK) plasma membrane (blue) and chloride ions (green) are shown. The channel is activated by protons binding to sites within the extracellular domain. Sodium and calcium ions enter the protein complex through fenestrations at the protein/plasma membrane interface (red arrow) and pass through the pore into the cell. This leads to membrane depolarization and downstream cellular effects. Amiloride [79] and PcTx1 [80] bind to a site near the interface of two subunits in the extracellular domain. Guanidino compounds [46] may interact at an extracellular site along the channel’s midline. The pore blocking sites for amiloride [79] and potential sites for diminazene [81] and memantine [82] are located within the channel pore, near the channel’s selectivity filter. The three ASIC1 subunits make a trimeric ASIC1a complex. The function of the channel can also be modulated by intracellular regulators, such as p11 [83].

## ASICs in Neurological Disease

ASICs have been postulated as having a role in a variety of neurological diseases, including Parkinson’s disease, stroke, traumatic brain injury, multiple sclerosis, and epilepsy. Although most of the data suggesting a role for ASICs in these conditions comes from preclinical studies and animal models of human disease, their possible contribution to these diseases can provide translational insights into how they mediate cell damage and clinical syndromes.

In neurodegenerative conditions such as Parkinson’s and Alzheimer’s disease, mitochondrial and microglial ASICs (primarily ASIC1a) may be involved in mediating cell death [84, 85]. ASICs may also contribute to stroke, although through a variable role in the cell response to cerebral ischemia, with ASIC1a activation (presumably from acidic hypoxic tissue) leading to cell death and ASIC2 being possibly protective [24]. Multiple sclerosis (MS) is another neurological disease with a potential ASIC contribution, as ASIC1a has been shown to be upregulated in a neuroinflammatory model, and could be responsible for axonal injury [86]. Finally, intense neuronal and synaptic activity in epilepsy causes reduced pH which can potentially activate ASICs and lead to seizure termination via an ASIC1a mechanism [29].

The potential contribution of ASICs to these wide-ranging neurological conditions, from neurodegeneration and excitotoxic/hypoxic cell death following ischemia and frequent synaptic activity in epilepsy, suggests that these channels may become pharmacological targets in human disease. Their wide expression and role from the cortex to deeper subcortical structures in these various conditions suggests that novel pharmaceutical agents against these channels would need to be designed to target specific ASIC subtypes under specific conditions, as there may otherwise be unwanted adverse effects associated with broad antagonism, particularly with CNS penetrant agents. Perhaps for migraine, peripherally acting agents, at the level of the trigeminal ganglion or sensory afferents (discussed below), would be sufficient for disease control.

## Potential Mechanisms of the ASIC Role in Migraine

### Meningeal Nociceptor Activation

Migraine pain involves the activation of dural nociceptors peripherally which then transmit pain for processing centrally [9]. Increased ASIC1a expression has been found in the spinal dorsal horn neurons following peripheral inflammation, and antagonizing this channel reduced pain-related behavior in rodents [22, 58]. Psalmotoxin 1 (PcTx1), a tarantula toxin, blocks the ASIC1a channel [87] and inhibits ASIC1a/2b current [41]. When administered intrathecally or intraventricularly, it reduced acute pain and pain-related behavior in two chronic pain models in rats [31]. Mambalgin-1, another ASIC1a antagonist but from the venom of a black mamba snake, also reduced pain behaviors in a rodent model [88]. Studies have shown that mambalgins inhibit homomeric ASIC1a, homomeric ASIC1b, and heteromeric ASIC1a + ASIC2a, ASIC1a + ASIC2b, and ASIC1a + ASIC1b receptors [89]. The heteromeric ASIC1a + ASIC2 receptor decreased acute pain and inflammatory pain in the rodent model, which was resistant to reversal by naloxone, suggesting a nonopioid analgesic effect [88]. Despite the ASIC expression and nociceptive role here being primarily spinal, there may be some role higher in the trigeminal pathway (see “New Animal Studies” section below).

In the peripheral nervous system, ASICs are expressed on sensory neurons responsible for signaling pain [23]. ASIC3 is one of the major ASICs expressed peripherally and is involved in pain originating from visceral organs such as the heart [90] and colon [59]. ASIC3 antagonists have been shown to reduce pain associated with cutaneous inflammation and pain arising from joints and muscles [45, 91].

Given that in nociceptive models, ASICs can sense acidic extracellular pH and cause pain as a result, it is feasible that this mechanism may be involved with the sensing of reduced meningeal pH in migraine [92]. Historical animal studies have demonstrated that dural afferents respond to acidic pH [93–95]. It has also been shown that dural stimulation leads to vasodilatation and therefore afferent trigeminal nucleus caudalis (TNC) activity, which can be blocked by amiloride, a nonselective ASIC antagonist [96]. Additionally, dural acidity leads to calcitonin gene-related peptide (CGRP) release from dural and trigeminal cell bodies and the release from trigeminal cell bodies can be blocked by a specific ASIC3 antagonist [97], suggesting a potential ASIC3-mediated role in migraine.

There is a clear role for ASICs in mediating dural afferent activity, especially since studies have shown that 80% of dural afferents will produce ASIC-like currents in response to acidic pH [98]. When low pH is directly applied to the dura of awake animals, they display headache-related behaviors which can be blocked at lower pHs by amiloride or at higher pHs by APETx2 (natural venom toxin from the sea anemone *Anthopleura elegantissima*, an ASIC3 antagonist) [94].

The mechanisms by which decreased dural pH may come about during the migraine process are unclear. The idea of neurogenic inflammation (a sterile meningeal inflammatory process involving several neuropeptides) occurring during migraine is a debated area [99]. However, mast cell degranulation within the dura can activate and sensitize dural afferents [100], and this degranulation is triggered by stress and CGRP release, following infusion of the nitric oxide (NO) donor nitroglycerin (NTG), and with increased estrogen [99]. Interestingly, these factors are all well-recognized potent migraine triggers in humans and one of these triggers, NO, has been shown to directly modulate ASIC activity. Cortical spreading depression (discussed below) can also lead to mast cell degranulation [101]. Mast cell granules contain acidic contents [102], and mast cell degranulation leads to the release of various proinflammatory cytokines such as interleukin-6 [103] and TNF-alpha [104], which can increase the activity and sensitivity of dural afferents to this reduction in pH surrounding the afferent nerve endings.

### Cortical Spreading Depression (CSD)

Migraine is a complex disorder, which causes nonpainful symptoms including aura in a proportion of sufferers [105]. Aura is characterized by a reversible neurological disturbance, which may accompany a migraine attack, or may occur in isolation in the absence of headache. It has been established in the literature that the electrophysiological correlate of migraine aura is cortical spreading depression or CSD [106], which consists of a wave of neuronal excitation of the cortex, followed by a more prolonged process of neuronal depression [107]. The process of CSD leads to ion flux, neurotransmitter release, and changes in cerebral blood flow and oxygenation levels [108, 109]. It is therefore feasible that acid-sensing ion channels may be involved in meditating this phenomenon.

It is unclear how CSD is related to the pain mechanisms in migraine, as subcortical areas and the brainstem seem to be vital to the pain of the migraine attack [9], yet the connection between CSD and such areas is disputed. Only a small proportion of migraineurs experience aura, so there are likely additional mechanisms involved in mediating migraine headache, unless CSD is clinically asymptomatic in the majority of migraineurs. Indeed, CSD can occur in other conditions and lead to aura-like symptoms in the absence of pain, including following traumatic brain injury, subarachnoid hemorrhage, epilepsy, and stroke [110], suggesting that CSD may be a process which is not immediately connected to the trigeminal pain network in migraine, but happens to be more common in migraineurs.

Preclinical studies have shown that CSD can cause cortical blood flow changes, as well as neuronal activity within the TNC, an area where trigeminal afferent input from the face converges in the brain [111]. It has also been shown that CSD can cause increased trigeminal ganglion activity, as well as increased activity in the C1 to C2 part of the trigeminal nucleus [112, 113], suggesting that CSD may lead to activation within the trigeminovascular pathway and therefore lead to migraine headache. It has also been found that the release of high-mobility group box 1 (HMGB1) from cortical neurons could link CSD leading to meningeal afferent activity [101, 114]. Additionally, connections between the cortex and the TNC, a brain area vital in migraine, can modulate TNC activity, providing a mechanism by which cortical activity could impact TNC activity and therefore pain signaling through the trigeminal system [115]. However, in animal models, CSD models are not able to produce behavior consistent with head pain [116–118]. It therefore remains unclear what connection if any CSD has with pain in migraine.

It has been suggested that a reduced pH from CSD could lead to the propagation of the CSD across a cortical area [119, 120]. This reduced pH could arise from hypoxia or ischemia from vessel changes associated with the phenomenon. A study in 2006 demonstrated the ability of amiloride (a nonspecific ASIC blocker) and psalmotoxin 1 (PcTx1), a blocker at the ASIC1a and ASIC1a/2b receptors, to inhibit CSD in a needle-prick rat CSD model. This effect was reduced in ASIC1 (−/−) knockout mice compared with wild type. Amiloride was also able to reduce cerebral vasodilatation in the rat model in response to electrical stimulation over the cranium, as well as to reduce electrophysiological responses to dural stimulation. As a result of this study, the authors gave amiloride, which is a licensed diuretic drug in humans, to 7 patients with treatment refractory migraine with aura and monitored them for clinical efficacy. Amiloride was able to reduce aura and headache symptoms in 4 out of 7 patients with otherwise intractable aura, suggesting a possible role for ASIC1 in migraine despite the nonselectiveness of amiloride for these channels [96].

### The Hypothalamus

The role of the hypothalamus in migraine is being increasingly appreciated, following animal studies [121–123] and more recent functional imaging studies in humans [124–127]. Its role in cluster headache has been more historically established, given the cyclical nature of the disorder [128], and this role formed the basis of the research into neuromodulation and deep brain stimulation of the hypothalamus as a treatment modality for cluster headache [129–135]. It has been hypothesized that the hypothalamus could be involved in mediating some of the premonitory and endocrinological disturbances which can accompany a migraine attack, such as yawning, thirst, and altered sleep patterns, through various connections and neuromodulators and neurotransmitters such as orexin and somatostatin [9]. It is also well recognized that alterations in sleep patterns can trigger migraine, and this association may be mediated by the hypothalamus [122]. Serum levels of hormones investigating the hypothalamic–tuberoinfundibular system (prolactin, growth hormone), the hypothalamic–hypophyseal–adrenal axis (cortisol), and pineal gland function (melatonin) were studied and shown to be altered in chronic migraine [136].

It is also clear from animal studies that the connections from the hypothalamus terminate in recognized regions of the trigeminovascular pain pathway. There are ascending and descending connections between the hypothalamus and the trigeminocervical complex (TCC), the periaqueductal gray (PAG), the rostroventral medulla (RVM), the nucleus tractus solitarius, and the nucleus raphe magnus [137], as well as the superior salivatory nucleus (SSN) [138]. Through these various connections and brain areas, the hypothalamus is able to convey ascending nociceptive input to higher structures for processing and also to modulate descending input.

ASIC3 is expressed throughout the hypothalamus [50], and ASIC1- and ASIC3-like currents have been demonstrated in hypothalamic neurons in culture [139]. Hypothalamic neurons also express the mRNA for various ASIC subtypes [140, 141]. Recently, it has been demonstrated that ASIC1a is expressed in lateral hypothalamic orexinergic neurons [142] and can modulate respiratory drive in a way that is blocked by ASIC antagonists. Orexinergic drugs have been researched in early trials for migraine treatment given the association between migraine and sleep and the postulated role of the hypothalamus in migraine [143]. Given the channel expressions within the hypothalamus, and the potential impact on hypothalamic function via orexinergic neurons, there is the suggestion that ASICs may play some role in migraine via the hypothalamus.

## The Emerging Role of ASICs in Migraine

We have discussed above the potential ways in which ASICs could be involved in the migraine process. The majority of the data presented has been preclinical, and apart from the small open-label amiloride study in seven subjects [96], we have limited supportive human evidence for ASICs playing a role in migraine, despite convincing animal studies.

### New Animal Studies

Most of the work that has been done on ASICs in migraine has focused on ASIC1, namely, ASIC1a, because of its widespread CNS expression. Emerging work which is currently unpublished but abstracted has used the demonstrated ASIC3-expressing neuronal projections to the TCC to study the role of ASIC3 inhibitors in trigeminovascular nociception. Trigeminal afferents were activated via middle meningeal artery stimulation, and extracellular recordings from the TCC were carried out. The authors demonstrated that APETx2 was able to reduce nociceptive firing in the TCC significantly more than saline with a prolonged 60-min effect at a 100-mcg/kg dose but not at a lower dose [144]. This data suggests that ASIC3 may also be a target for migraine therapeutics.

Additional indirect support for the role of ASIC3 in migraine comes from another study in which the authors tested for the expression of ASIC1, ASIC2a, and ASIC3 in the trigeminal ganglion of rats and that there was upregulation when the rats were exposed to a formalin orofacial pain inflammatory model. Migraine can involve facial pain, and indeed can present with facial pain alone. This upregulation could be reversed with pharmacological ASIC blockade and with genetic deletion of ASIC1 [72]. This is supported in another study which showed a positive correlation between periodontal ASIC3 expression in rats following experimental tooth movement (which was conducted through springs and deemed painful on a rat grimace score) [145]. This evidence is supportive that ASIC3 may be involved broadly in trigeminal pain. Additionally, a study in 2013 showed that ASIC3 is the predominant ASIC responding to a reduced meningeal pH and in the context of inflammation; dural afferents are responsive to even smaller pH changes within the meninges, suggesting a role in headache [94]. These effects can be blocked with ASIC3 antagonism.

Further, it has recently been shown that the dural pH necessary to cause migraine-like behavioral responses is substantially higher if animals are first sensitized, or “primed,” by prior exposure to a noxious dural stimulus. In this study, cytokine interleukin-6 (IL-6) was applied to the dura and following resolution of the initial IL-6 behavioral responses, animals were then sensitive to stimulation of the dura with a pH 7.0 solution. Even after sensitization, the responses to dural pH 7.0 were blocked by APETx2 indicating a role for ASIC3. Control animals did not respond to dural pH 7.0, which demonstrates that the meninges can be sensitized to modest pH changes after a priming event. These findings suggest that ASIC-dependent signaling from the meninges is likely to be even more important in the context of sensitization, such as that occurring during the development of a migraine attack or even potentially in migraine patients between attacks [146] .

### Human Imaging Work

Acid–base changes within the brain can be detected using modern brain imaging, namely, MR proton spectroscopy. This has been used in migraine, in a hypoxia *versus* sham triggering model [147]. It has been known for some time that hypoxia has the potential to trigger migraine attacks [148, 149], and it has been demonstrated that hypoxia can induce CSD in mice and that the threshold to CSD is lower with increased CSD duration in response to potassium [150]. In healthy volunteers, hypoxia is associated with increased brain lactate [151], and this rise in lactate in association with hypoxia has also been shown in migraine with aura interictally [152]. The authors of this most recent migraine spectroscopy study therefore aimed to study the ability of hypoxia to trigger migraine with visual aura attacks; they subsequently used imaging and blood tests to measure the visual cortex concentrations of glutamate and lactate and serum metabolites in response to hypoxia *versus* sham in both migraine with aura and healthy controls. In both population groups, hypoxia caused increased visual cortex lactate and dilatation of the cranial vasculature, and in the migraine with aura group, hypoxia triggered aura attacks in 7 subjects out of 15. The serum lactate was increased after hypoxia more in patients than in sham and in controls. The authors hypothesized that in migraine with aura, hypoxia may lower the threshold for CSD via lactate increases, and cause prolonged CSD. It is unclear how the increase in lactate is mediated, be it through mitochondrial dysfunction or other mechanisms. The brain responses to lactate may be mediated through ASICs, but more work is clearly necessary to address this possibility directly.

This demonstrated increase in brain lactate in response to a recognized migraine trigger suggests again that ASICs may be involved in mediating the response to lactate and therefore in mediating CSD and possibly other migraine mechanisms. Two interictal MRI studies have found increased brain lactate in patients with migraine with aura [152, 153] in small patient groups using 1.5-T MRI, and such findings have not been reproduced at 3 T in migraine without aura [154, 155]. Additionally, a recent MRI study has demonstrated an undisrupted blood–brain barrier during spontaneous migraine with aura [156], and another study has demonstrated that a potent anti-migraine drug, dihydroergotamine (DHE), does not bind in the brain when administered in migraine without aura, suggesting that the main effect is outside of the blood–brain barrier and that the blood–brain barrier remains intact during triggered migraine attacks [157]. These studies suggest that metabolite changes during CSD do not cause blood–brain barrier changes, and are therefore unlikely to cause the brainstem changes that occur before and during migraine headache, and that effective anti-migraine treatments do not necessarily need to penetrate the blood–brain barrier to be efficacious.

## ASIC Pharmacology and Reference to Migraine

In recent years, awareness has increased about the pharmacology of ASICs and potential novel spider and venom toxins which have proved useful in experimental research to understand the role of these channels in disease. These studies are reviewed in detail in references [158, 159]. A selection of pharmaceutical agents targeting these channels is discussed here briefly, in particular those with clinical significance for human disease.

Amiloride, which is a diuretic agent which spares potassium, was the first recognized blocker of ASICs, with broad anti-ASIC action with no differentiation between channel subtypes [74, 160]. At higher concentrations, amiloride is able to have the paradoxical effect of opening ASIC3 homomeric and heteromeric channels at a normal pH, and enhances the channel activation in response to mild acidosis [161]. This activity is possibly mediated at a central site between the three extracellular domains of ASIC subunits where other guanidine-containing compounds have shown efficacy [46, 162].

A-317567 is another ASIC antagonist that affects ASIC1a, 2a, and 3. Furthermore, this antagonist has shown higher affinity for ASIC1a and ASIC3 compared with ASIC2a [163]. Additionally, this compound may have non-ASIC effects suggesting that other may protein targets can interact with the compound. In animals that received A-317567 in pain studies, sedation was observed. Sedation is not thought to be an ASIC-mediated effect, suggesting that the A-317567 compound interacts with other central nervous system receptors and has the potential for unwanted side effects [164].

Several categories of drugs that are classically used for pain can have actions at ASICs. Nonsteroidal anti-inflammatory drugs (NSAIDs) have anti-ASIC1a and ASIC3 properties [165]. Diclofenac and ibuprofen antagonize ASICs expressed in rat hippocampal interneurons [166]. However, these anti-ASIC1a and ASIC3 effects are at higher concentrations than the effects observed with lower concentrations of amiloride. Whereas most local anesthetics exert their anti-nociceptive effect through sodium channels, some have been shown to have anti-ASIC properties, including tetracaine on ASIC3 [167], lidocaine on ASIC1a [168], and the general anesthetic propofol on ASIC1a and ASIC3 [169].

Clinically approved and available compounds for other diseases may also have ASIC modulatory effects. One example is the drug memantine, a hydrophobic monoamine that targets ionotropic glutamate receptors for treating dementia associated with Alzheimer’s disease [82]. Memantine inhibits ASIC1a in both neurons and cultured cells [170]. Furthermore, memantine was shown to antagonize the channel at hyperpolarized voltages but potentiated (increased) ASIC1a-mediated current at zero voltage [82]. Modeling data suggests that memantine blocks the channel deep within the ASIC1a pore. However, the site within ASIC1a responsible for potentiation by memantine remains unclear.

Finally, there are compounds that perhaps surprisingly have effects at ASICs, and these effects may not play a role in their primary mechanism of action. For example, the anti-protozoan compound diminazene exhibited anti-hyperalgesic activity in models of chronic inflammatory pain [171]. Diminazine acts as a channel blocker for ASICs [81, 171], and although this action may contribute to its efficacy for pain, it is unclear whether this mediates its effects on protozoa. Nonetheless, this example illustrates the possibility that modulators of ASICs will be discovered through screening of currently existing compounds that are used for other purposes.

## ASICs in Migraine Drug Development

We have outlined in this review the preclinical and clinical evidence supporting the role of ASICs in migraine. From the evidence presented so far, it is likely that there are two predominant roles, 1) in the modulation of CSD and therefore migraine aura, in particular with the evidence supporting ASIC1a, and 2) in the modulation of nociceptive signaling from the meninges, in which there is the emerging role for ASIC3 in trigeminovascular nociception. It is clear that this is an evolving field, and more understanding and research is needed to understand the various ASIC subtypes and their potential roles in human disease.

Unfortunately, there are currently no ASIC subtype-specific antagonists which are available for safe use in humans, and the use of amiloride is limited by its lack of specificity; it has actions at non-ASIC receptors, as well as broad ASIC antagonism. However, understanding brain metabolism during migraine attacks using functional imaging, and further imaging of aura in migraineurs, may provide us with additional understanding of how changes in pH and subsequently ASIC activity may contribute to CSD. Additionally, further preclinical models of both aura and trigeminovascular activation and use of ASIC subtype-selective antagonists are likely to provide further valuable insights into the mechanisms by which these channels may be involved in migraine headache.

As discussed earlier, their wide CNS and PNS expression makes ASICs attractive therapeutic targets. However, simultaneous disruption of various channel subtypes may lead to unexpected effects. Targeted therapies (perhaps against ASIC1a and ASIC3) are likely to be more useful and yield fewer unwanted adverse effects. Peripherally acting agents may be sufficient in migraine, given recent evidence from the DHE study which suggests no or limited blood–brain barrier permeability of this efficacious abortive in migraine therapy [157], and the likely peripheral sites of action of the novel anti-calcitonin gene-related peptide (CGRP) therapies, which have shown clinical efficacy both acutely and preventively and are in late-phase clinical trials [172].

## Conclusions

We have discussed here the broad range of preclinical and clinical research which supports a potential role of ASICs in nociception, as well as other neurological conditions. There is increasing evidence for their role in migraine, and it is likely that further animal work, human genetics, and functional imaging in migraine patients may help us refine the contribution of these channels to the disorder. In an era in which there is an ever-increasing need for effective therapies for migraine, which is a disabling condition usually affecting otherwise fit and well young individuals, ASICs provide an attractive potential therapeutic target, perhaps particularly so in migraine with aura. Their role in migraine, once better understood, could well be extrapolated to other CNS conditions which may share some common mechanisms, such as symptomatic CSD following traumatic brain injury or stroke, providing a useful treatment strategy for these conditions also.

## Electronic supplementary material

ESM 1 (PDF 1.19 mb)
